# Challenges to effective and autonomous genetic testing and counseling for ethno-cultural minorities: a qualitative study

**DOI:** 10.1186/s12910-020-00537-8

**Published:** 2020-10-15

**Authors:** Nehama Cohen-Kfir, Miriam Ethel Bentwich, Andrew Kent, Nomy Dickman, Mary Tanus, Basem Higazi, Limor Kalfon, Mary Rudolf, Tzipora C. Falik-Zaccai

**Affiliations:** 1 The Azrieli Faculty of Medicine, Bar Ilan University, 8 Henrietta Szold St, P.O. Box 1589, Safed, Israel; 2 The Institute of Human Genetics, Galilee Medical Center, Nahariya, Israel; 3 Division of Hematology and Oncology, University of Colorado Hospital, Aurora, Colorado USA; 4 Israeli Ministry of Health, Akko District, Acre, New York USA

**Keywords:** Prenatal genetic testing, Ethno-cultural minority, Genetic counseling barriers, Qualitative research, Multicultural society, Arab minorities

## Abstract

**Background:**

The Arab population in Israel is a minority ethnic group with its own distinct cultural subgroups. Minority populations are known to underutilize genetic tests and counseling services, thereby undermining the effectiveness of these services among such populations. However, the general and culture-specific reasons for this underutilization are not well defined. Moreover, Arab populations and their key cultural-religious subsets (Muslims, Christians, and Druze) do not reside exclusively in Israel, but are rather found as a minority group in many European and North American countries. Therefore, focusing on the Arab population in Israel allows for the examination of attitudes regarding genetic testing and counseling among this globally important ethnic minority population.

**Methods:**

We used a qualitative research method, employing individual interviews with 18 women of childbearing age from three religious subgroups (i.e., Druze, Muslim, and Christian) who reside in the Acre district, along with focus group discussions with healthcare providers (HCPs; 9 nurses and 7 genetic counselors) working in the same geographical district.

**Results:**

A general lack of knowledge regarding the goals and practice of genetic counseling resulting in negative preconceptions of genetic testing was identified amongst all counselees. Counselors’ objective of respecting patient autonomy in decision-making, together with counselees’ misunderstanding of genetic risk data, caused uncertainty, frustration, and distrust. In addition, certain interesting variations were found between the different religious subgroups regarding their attitudes to genetic counseling.

**Conclusions:**

The study highlights the miscommunications between HCPs, particularly counselors from the majority ethno-cultural group, and counselees from a minority ethno-cultural group. The need for nuanced understanding of the complex perspectives of minority ethno-cultural groups is also emphasized. Such an understanding may enhance the effectiveness of genetic testing and counseling among the Arab minority group while also genuinely empowering the personal autonomy of counselees from this minority group in Israel and other countries.

## Background

In the last decade, significant developments in genetic technology have expanded the breadth of potential prenatal diagnoses for numerous genetic conditions and prenatal genetic testing. Consequently, genetic counseling has become increasingly intricate for both genetic counselees and healthcare providers (HCPs), such as genetic counselors. HCPs working in the field of human genetics and specifically genetic counselors are expected to communicate complex genetic information while operating under an obligation for patient autonomy to help patients make informed choices free of the influence of the HCP’s value system [1–3].

This approach acknowledges people’s tendency to make decisions based on their social and personal backgrounds—namely, decisions affected by personal and cultural preferences, not only by medical considerations [4, 5]. At the same time, the literature acknowledges that people from minority cultural groups, including ethno-cultural groups, tend to underuse genetic testing and counseling services [6–9].

Against this backdrop, a growing importance has been attributed to defining cultural competence in genetic testing and counseling. Thus, cultural competence is broadly understood as acknowledging and incorporating “the importance of culture, assessment of cross-cultural relations, vigilance toward the dynamics that result from cultural differences, expansion of cultural knowledge, and adaptation of services to meet culturally unique needs” ([10], p. 294). Therefore, the need for in-depth understanding about social and cultural influences on patients’ health beliefs and behaviors, especially individuals from minority cultural groups, has become paramount to increasing their utilization of genetic testing and counseling while respecting and possibly empowering their autonomy [11–13]. Indeed, strategies to improve cultural competence are an active area of research in the context of genetics [12, 14–20]. Cultural competence in the context of genetic counseling services is tied to respect and empowerment of autonomy. The aim of culturally competent-informed counseling is not to induce the use of genetic testing against the will or choice of an individual counselee, but rather to make genetic testing accessible to various cultural groups and assist their members in making their *own informed choices* about genetic testing. In fact, the emphasis on counselees’ autonomy follows current standard guidelines for genetic counseling as they were formulated by the Genetic Counseling Definition Task Force of the National Society of Genetic Counselors (NSGC) [21].

However, interestingly, the possible interaction between genetic counselees from a minority cultural group and HCPs, such as genetic counselors from the majority cultural group, has been less studied. Moreover, the idea of a minority cultural group may be more fluid than currently represented in the literature about genetic testing and counseling. For example, counseling a minority group may be associated with a specific ethnicity, but culturally and religiously, an ethnic group may be comprised of multiple religious faiths, which may have a cultural impact on the manner with which genetic counseling, genetic screening, and abortions are perceived.

In this context, Israel may provide a particularly interesting test case. To begin with, Israel has evolved as a leader in genetic research and genetic testing services, particularly with respect to next-generation genetics manifested in genetic testing and tools such as prenatal genetic diagnosis (PND), pre-implantation genetic diagnosis (PGD), and pre-implantation genetic screening (PGS) [22–26]. Yet previous studies have already indicated that the Arab minority ethnic group, accounting for approximately 25% of Israel’s entire population, tends to underutilize genetic counseling and the use of the aforementioned genetic testing tools [27, 28]. At the same time, a previous study conducted among the Arab population found that religious reasons preventing people from performing abortions was indicated by fewer than 10% of the respondents as underlying their refusal to certain genetic screening [28]. This finding seems to provide support for the idea that the underutilization of genetic counseling and testing services in the Arab population does not reflect a clear cultural stance against using these services. Rather, more nuanced cultural barriers may exist, thereby underscoring the need for better understanding and efforts to address these barriers.

Furthermore, rates of birth defects are higher in the Arab population than in the Israeli Jewish community [29, 30]. Thus, effective and culturally tailored genetic counseling is of paramount importance to this population. In fact, one study pointed out that Arab patients’ expectations of instruction on how to proceed and the low level of genetic literacy among the public generate a cultural misconception. Referral to a genetic clinic is perceived as resulting in direct advice on issues such as the choice of a marriage partner, reproductive options, and selective abortion of an affected fetus [31]. Hence, contrary to the professional commitment of HCPs involved in genetic testing and counseling to respect the autonomy of counselees, prior studies suggest that Arab patients might perceive these services as curtailing their autonomy.

Meanwhile, in specific geographical regions in Israel,, such as the northern part of the country, the Arab population comprises between 50 and 60% of the population [32]. In the Western-Galilee Acre region, Arabs make up as much as two-thirds of the population. Consequently, although united under a common Arabic language, the Arab population in this region is particularly heterogeneous—namely, composed of different religious-cultural subsets (primarily Muslims, Druze, and Christians) with different religious beliefs and living in culturally unique and socially isolated villages and tribes [33].

Therefore, the current Israeli case study may assist in gaining a better understanding of the nuanced cultural underpinnings and interactions between the Arab minority and the majority groups in Israel and the aforementioned regions, possibly affecting perceptions about genetic counseling service that result in the underuse of this service. Furthermore, genetic counseling in Israel follows the aforementioned guidelines for genetic counseling as they were formulated by the NSGC, including their emphasis on respecting and empowering the counselees’ autonomy. Hence, addressing the underuse of genetic counseling and testing services should clearly not be accomplished by forcing the individual counselee to utilize these services. Indeed, in Israel, genetic screening for carriers of genetic disorders is performed on a voluntary basis; there are no sanctions against those who choose not to perform genetic testing, and there are no state-based rules requiring pre-married couples to undergo certain genetic screening (Cf: Iran, Cyprus) [34–37]. Rather, the emphasis is on the better understanding of possible culture-based barriers that may cause misconceptions of genetic counseling and testing, ultimately leading to their underuse by the Arab population in the country. This sort of understanding may also be applicable to other Western countries with multicultural groups (Arabs and others) facing similar challenges of underuse of genetic counseling and testing by minority multicultural groups despite being committed to counselees’ autonomy and right to choose.

## Methods

### Study design

We conducted a qualitative study to address two main research questions: (1) What are the opinions, experiences, and preconceptions regarding genetic testing and counseling among the three predominant Arab populations in Israel (Christian, Druze, and Muslim)? (2) How do genetic counselors and nurses in mother and infant clinics describe the unique challenges of working with this population in the context of genetic counseling? This study is part of an action research study to identify challenges pertaining to genetic counseling of minority populations and subsequently implement interventions that address these issues. This study was approved by the Ethics (Helsinki) Committee of the Israeli Ministry of Health [approval #0118–14], and all participants signed an informed consent form prior to participating in the study.

### Study participants

For the individual counselee interviews, our study participants consisted of 18 women (Table 1) selected through purposive sampling from three different Arab cultural groups (Muslims, Druze, and Christians) representing inhabitants from two distinct villages (one homogenous and one heterogeneous village) for each cultural group. The selection criterion was women of childbearing age who came for their routine medical follow-up at the mother and infant clinic in their villages.

**Table 1:** Participant characteristics

	Druze (6)	Christian (6)	Muslim (6)	Total (18)
Mean age (years)	28.8	28.8	26.5	28
Homogenous village	3	3	3	9
Heterogeneous village	3	3	3	9
Consanguinity	3	2	1	6
Considers herself religious	5	1	4	11
Education- high school (+)	5	6	6	15
Academic		2		
Occupation- Housewife	4	1	6	11
Pregnancy	2	4	4	10
Referral to genetic counseling during pregnancy	3	3	2	8
Received genetic counseling	1	3	1	5
Focus group - nurses	3	3	3	9
Focus group- genetic counselors			2	7^a^

^a^ (5 Jewish genetic counselors)

The nursing focus group consisted of nine nurses from the three different ethno-cultural groups working in mother and infant clinics in various villages in the Galilee. These nurses actively guide women in the community during pregnancy, labor, and subsequent child development. The selection criteria for this focus group in the study were, therefore, nurses working in mother and infant clinics in the Galilee who were affiliated with any one of the three main Arab cultural groups (Druze, Christians, and Muslims) and expressed interest in the project.

The seven genetic counselors who participated in the focus group worked in genetic departments in four hospitals in Northern Israel. The inclusion criteria for this focus group were counselors who worked in the four hospitals’ genetic departments and who had at least 5 years of genetic counseling experience for varied ethno-cultural groups, including the Arab ethnic minority.

### Data collection

Interviews lasting 1–1.5 h were conducted at mother and infant clinics in separate rooms to ensure privacy. They were carried out in Hebrew and Arabic and utilized a semi-structured interview strategy. Demographic data were collected via a standard questionnaire prior to the start of each interview. The interview utilized fictional vignettes regarding birth defects, prenatal diagnoses, and termination-of-pregnancy options to elicit the interviewees’ viewpoints regarding genetic counseling (see Construction of the Interview Guide). For the risk communication analysis, two hypothetical scenarios were presented to the participants. First, participants were asked to place a mark anywhere along a horizontal line (from 0 to 100% chance) to indicate their perceived risk regarding amniocentesis [38]. For the second test, participants were asked to indicate along a second horizontal line their perception regarding the relative risk of 1:200 for Down syndrome (see Supplementary material).

### Construction of interview guide

The first author, an experienced genetic counselor, drafted several common scenarios revolving around reasons for referral to genetic counseling and relating to risk assessments, possibilities of prenatal diagnosis, and possibilities of the termination of pregnancy. These scenarios were further discussed and enhanced with the last author, who is the head of the genetic counseling unit at one of the participating hospitals. Ultimately, three different scenarios were chosen:

1. Congenital heart defect during pregnancy as a trigger for discussion of risk assessment and prenatal diagnosis
2. Family history of developmental impairment as a trigger to the question of etiology for genetic counseling and risk assessments for birth defects
3. Family history of hereditary disease as another trigger for the issue of prenatal diagnosis and the possibility of termination of pregnancy

Focus groups were conducted, as previously reported, in a group dynamic to stimulate discussion, gain insights, and generate ideas to pursue a topic in greater depth [39]. The primary question that was used to initiate the discussion was: “In your experience, what are the unique challenges and/or difficulties (if any) in providing effective genetic counseling to the Arab population?”

### Data analysis

All interviews, both individual and with the focus groups, were audio-recorded by the first author and transcribed verbatim for subsequent analysis by a professional transcription service (Mao’f Inc.). Grounded theory content analysis was performed on the transcribed text to identify key themes that emerged from the participants’ unique perspectives [40, 41]. The process of content analysis involved the following steps. Data were coded line-by-line by the first author using an inductive coding method [42]. A coding tree was developed, and codes were gathered into main themes and subthemes using the constant comparative method, by which researcher(s) develop concepts from the data by simultaneously coding and analyzing the information [43]. Coding continued until saturation was reached—namely, no more new themes were identified during analysis. The first writer who handled the core coding of the data into themes and subthemes noted that, when conducting the analysis of the last three individual interviews, no new main themes or subthemes emerged. To ensure the trustworthiness of the data analysis, the first and second authors discussed the coding and the emerging themes and subthemes throughout the analysis process for interviews and focus groups. We chose this method of analysis rather than two separate coders independently analyzing the entire dataset because, in our research group, only the first author had specific expertise in genetic counseling, which was the core focus of the study. We further discuss the issue in the Study Limitations subsection.

The discussions regarding the analysis of the interviews included a 4-step process. (1) The first author conducted an initial coding of the data for either one interview at a time (for the first half of the interviews under analysis) or three interviews (for the second half). This coding was done using a table that indicated the themes and subthemes that have emerged and highlighting the relevant text in the interview from which these themes and subthemes were derived. (2) The second author read and made written comments pertaining to instances where she had questions regarding the analysis of the first author. (3) Both authors discussed the questions raised by both authors regarding the analysis. In cases where the questions regarding the analysis turned into disputes about how to interpret the text, both authors went back, simultaneously, to the original transcript and examined the larger context from which the particular disputed fragment was taken until reaching an agreement. (4) At the end of their discussion, the first author updated, as necessary, both the interpretation table and an overall codebook table for the interviews so that they would reflect the most updated and agreed-upon coding.

## Results

### Participant characteristics

Table 1 provides general characteristics for the interviewed counselees, all women with a mean age of 26–28 years. Almost half had previously been referred to genetic counseling services. The nursing focus group consisted of women as well, who were evenly distributed among the ethnic groups. All of the nurses were fluent in Hebrew and Arabic. The counselor focus group was predominantly Jewish (5/7 counselors), but also included two Muslims; their ages ranged between 30 and 45 years. One counselor was male.

### Lack of pre-counseling knowledge but desire to learn more

Currently, potential genetic counselees are identified by a primary care physician in the community and referred to a genetic counseling service. Eight of the 18 participants (predominantly Muslim and Druze) stated that they had never heard of genetic counseling (Table 2, 1a). Most of the remaining participants had already been referred to genetic counseling. Of these, two stated that they only remembered signing some papers, but not what they were for, suggesting either a profound lack of explanation to the counselees regarding what genetic counseling is or a lack of comprehension and/or retention of the information provided. One of the Druze participants, who was referred to genetic counseling (GC) due to an inherited disease in the family, described her impression and recollection of a GC session:

*“And she asked me—if the fetus has a problem/a defect, will you terminate the pregnancy? I told her, of course not. That's my answer ... And then she gave me a paper to sign.”*

Another Muslim participant said:

*“Yes, I heard (about genetic counseling), I went once in the second pregnancy, because there was a problem for my daughter in the heart she had ‘Vias di’ [referring to VSD] … I don't know, I didn't like it, just asked me ... (about) the family, who's sick in the family, who's not sick ... That's how they asked me ...”*

Some participants did not understand the full extent of the risks for birth defects and genetic diseases. More than two-thirds believed that “normal” screening tests implied a healthy pregnancy, rendering further genetic testing and counseling unnecessary, and they did not appreciate the limited focus of screening or that other genetic risks are involved (Table 2, 1b). For example, one Muslim participant shared her understanding of genetic counseling in the following manner:

*“I went to the clinic, I took the blood tests, I asked her [the nurse] ‘Is everything okay?’, She told me everything is okay, no problem. If there is a problem or something, you would have been referred to a genetic counselor.”*

Another fundamental lack of understanding regarding the meaning of GC emerged in the response of a Druze woman:

*“I did another test. Which is like a page that lists all of my genes. And that's for all pregnancy I was told to keep it …”*

Altogether, these responses and comments imply a general ignorance or naivety regarding genetic risks and genetic counseling. Yet despite this preliminary lack of knowledge, overall, 7/18 counselees stated a desire for more access to knowledge and awareness about genetic testing and counseling opportunities (Table 2, 1c).

*“… Now I don’t understand so much [regarding genetic counseling]. If there was a lecture I would go [to it] ... the head of genetics ... he should go to the government and tell … tell them to do [lectures], that's very important. … It gives them knowledge. And I also want to expand this knowledge …” (Christian participant)*

**Table 2:** Thematic responses by participants according to ethnic sub-group

		Fraction of participants expressing the indicated theme			
Theme		Muslim	Druze	Christian	Total
1. Pre-counseling knowledge regarding genetics and genetic counseling	a) Had not heard of genetic counseling	4/6	3/6	1/6	8/18
	b) Confusion between genetic screening tests and genetic counseling	5/6	5/6	3/6	13/18
	c) Would expect increase awareness/understanding in community	2/6	3/6	2/6	7/18
2. Negative conceptions of genetic testing and counseling	a) Unnecessary (only results in more questions)	3/6	2/6	2/6	7/18
	b) Expect negative event and/or recommendation for termination of pregnancy	2/6	3/6	1/6	6/18
	c) Ultrasound is more reliable	3/6	2/6	0/6	5/18
	d) Folk stories of contradictions between genetic predictions and outcome of pregnancy	2/6	3/6	0/6	5/18
	e) Negative attitude toward counseling due to family and community influence	3/6	3/6	2/6	8/18
	f) Reassurance of healthy pregnancy	1/6	1/6	1/6	3/18
3. Family involvement	a) Heavily involved	3/6	1/6	0/6	4/18
	b) Sharing/supportive	1/6	0/6	3/6	4/18
4. Spousal involvement in decision making	a) Joint discussion	3/6	3/6	2/6	8/18
	b) Woman decides	3/6	4/6	5/6	12/18
	c) Man decides	1/6	1/6	0/6	2/18
5. Termination of pregnancy	a) Opposition due to conscience	3/6	2/6	1/6	6/18
	b) Opposition due to culture/religion	3/6	5/6	1/6	9/18

### Negative personal and cultural preconceptions about genetic counseling

Once introduced to the idea of genetic counseling, many participants stated a preliminary negative stance to the process for various personal and societal reasons. More than half expressed negative attitudes regarding the potential benefit of genetic testing and counseling. Specifically, seven participants denoted referrals to genetic counseling as “unnecessary” or “threatening,” often assuming a referral to be equivalent to a recommendation for abortion (Table 2, 2a, b). One Muslim woman explained:

*“Everyone who goes to genetic counseling has a 'black stain'… She went for genetic counseling… [which] means that there is something [wrong] with her son.”*

Another Muslim woman elaborated on the reason for her stance against GC:

*“I'm against [genetic counseling] ... Because, if they say everything is fine, to her and to the child, and a perfectly healthy child is born, so, a month later—then he is ill, then to throw him away?*

*“The genetic counselor will convince her to abort .... I don't know ... I'm against ... genetics ... I don't know why, I'm against … I want to tell you, I did not go ... [to genetic counseling], I have family, with deafness—my mother's nephew, but I didn't ... go to receive [genetic counseling].”*

Nearly one-third (5/18) of the participants regarded genetic testing and counseling as unnecessary, although an ultrasound was perceived to be reliable enough (Table 2, 2c). For example, when one of the Muslim women was asked why she did not go to GC, she responded as follows:

*“I did not know ... my mother-in-law ... she is a nurse, and I asked her [about going to genetic counseling] ... but [she said that it is] not so much needed, not urgent ... if you want, you can go. I don't know, I thought it was a conversation, questions, etc. No tests or anything like that.”*

A Druze lady offered her insights regarding the lack of need for GC, stating:

*“During the pregnancy, it does not matter, [it’s] enough to visit the doctor [physician], who claims that there is a problem and will take care after delivery .... So why is genetic counseling needed?”*

An important source of mistrust of genetic data was due to a folk narrative contradiction regarding prenatal results. Five of the 18 participants maintained that they knew community members who had been told their child would suffer from a major birth defect when, in fact, the child was born healthy (Table 2, 2d). Interestingly, neither of these two themes were expressed by the Arab Christian women who participated in the current study. In contrast, one of the interviewed Muslim women shared the following story from her larger family:

*“… I will give you an example, my cousin… when his mother was pregnant, they [i.e., medical staff] told her not to bring the child because he is ‘without a head.’ She did not agree and gave birth and now … I mean, next year, my cousin is going to get married. He has no problems.”*

Almost half (8/18) of the participants described negative attitudes toward the use of genetic services due to family members’ negative views (Table 2, 2e). One Druze participant quoted her father-in-law:

*“… Why should you go to genetic counseling? They will tell you to terminate the pregnancy, to put the child down. … You won't do that, so don't go ... why should you go?”*

A Muslim participant shared a story her mom told her regarding the circumstances in which the participant’s sister was born, implying the futility of GC:

*“I was also told [by] my mom ... like, we had an accident. I was little ... and she was pregnant, [in the] last month … and then she was told in the hospital that the baby, the fetus ... he is not [well] ... that he will come out ... unnatural … [the fetus] has something, he has a problem ... better to terminate [the pregnancy…] and so on [...] And then … it's my sister [who was born], and she's … not because she's my sister, she's beautiful [giggling], she is very smart.”*

Interestingly, only 3/18 of the participants anticipated the potential for a positive outcome or reassurance of a healthy pregnancy (Table 2, 2f). For example, one Muslim participant offered a more positive interpretation of the genetic counselor’s role.

*“… He [The GC] would sit with her, explain to her about her illness … and the results, help her mentally, reduce these things …”*

### Culturally based differences in decision-making about pregnancies between Arab subsets

With the identified issues in mind, we next wanted to determine if any subset of specific opinions or perceptions might influence the utilization or outcomes of genetic testing and counseling services. Several major areas of difference were identified.

Compared to other cultural subsets, Christian women were more familiar with genetic testing and counseling services (Table 2, 2a). In addition, the negative attitudes described herein were mentioned less often by Arab Christian women. None of them mentioned either ultrasound screening as the only reliable pregnancy examination or a folkloric contradiction between pessimistic medical predictions and pregnancy outcomes (Table 2). More Christian women mentioned the supportive role of their families and the option given to them to share their worries (Table 2, 3b).

One participant described the worries she shared with her sister during her sister’s pregnancy:

*“… And she had to do genetic counseling, because she was told there was a risk of a syndrome, mental retardation, or muscle disease, and they told her she had to have an abortion. … There was a 30% risk. ..The whole family [was] traumatized … and I, together with my parents, consulted [her] together and she finally decided that she wanted the baby…”*

Muslim women tended to report greater attempted family interference in decision-making regarding pregnancy decisions than Druze and Christian women (Table 2, 3a). As one participant said:

*“… It is very difficult, everything is forbidden, and they are different … the man decides … and his parents sometimes … I will not ask them, but they will usually interfere and it should not be in genetic counseling ...”*

Unique to the Druze culture, there was little reference to family involvement in prenatal counseling during the interviews. One of the nurses described the practice in the Druze community:

*“… everything stays in a kind of secret between us and the couple. ... The Druze women tend to keep their pregnancy decisions private.”*

Indeed, one of the Druze women stated:

*“For example, if there is anyone here, we will find her fetus, oh boy, God forbid, with ‘fanconi’ and they said there would be postpartum complications, … after birth, I recommend that, do not tell anyone … do what your conscience tells you. …”*

Despite this, the vast majority of the women, regardless of the particular ethno-cultural subset with which they were affiliated, stated that they—not their spouses—would ultimately make pregnancy decisions (Table 2, 4a, 4b).

- *“… My husband told me that, if there is a problem with the baby, we will terminate the pregnancy... I said no! Even though it caused me a lot of problems. ...” (Druze participant)*
- *“… Me too, you know, it is not only my decision, I have [a] husband, my husband’s parents … it is not only my decision. However, when I was pregnant, and they told me that he (the fetus) has heart anomaly, my husband told me he does not want (the pregnancy) … He wanted to terminate the pregnancy in case of ‘mongoly’ (derogatory slang for Down syndrome). I said I don’t want to terminate… In the end, I was the one to decide. ...” (Muslim participant)*
- *“You know, my mom, … she terminated a previous pregnancy. In my brother's pregnancy she refused and he was born naturally. ... My father ... at that time, [he] told her, do not terminate pregnancy, but she did...” (Christian participant****)***

Only two of the 18 participants stated that the father dictated the decision regarding a pregnancy (Table 2, 4c).

The choice to terminate affected pregnancies was also a key topic. Many Druze and Muslim women opposed the termination of a pregnancy. Almost all (5/6) Druze women and half (3/6) of the Muslim women said they opposed the termination of a pregnancy for cultural or religious reasons (Table 2, 5b), often using the word “*charam*,” meaning forbidden under religious law.

- *“I am not going to terminate a pregnancy (!) because ... [it is] not good to terminate a pregnancy (!) It is wrong to end someone's life … Even in our religion (Muslim), it is unacceptable ... haram ... if it was in the beginning, it is possible ... if it, there is a spirit child, and there are all these … then no ...” (Muslim participant*)
- *“Abortion, no. We… because in our religion it is forbidden to us. Oh, it is like you killed a person. According to your religion (Jew), your commandments, ‘not to kill,’ here too, no … even a fetus ...” (Druze participant)*

Only two out of the six Christian women interviewed stated opposition to the termination of a pregnancy due to either conscience or religion (Table 2, 5a and 5b).

- *“This is quite a bit of a dilemma. It is … by the way, until we are not in this position, we cannot decide, and I'm a mom and I understand that … really. This, it's not a decision ... if—in my head I say terminate the pregnancy, but in my heart I say ‘no’. What will I decide at the end? I don't know [smirking].”*
- *“It helps, but for me personally it doesn't help ... I'm not ready to terminate a pregnancy for nothing. I can’t, it is my fetus. … Everything is from God, ... I believe so … I believe in God, but I do not go to church ... But I believe there is a God ... There is a religion ... There are miracles ...”*

### Lack of effective communication regarding statistical data from counseling services

**Fig. 1:**
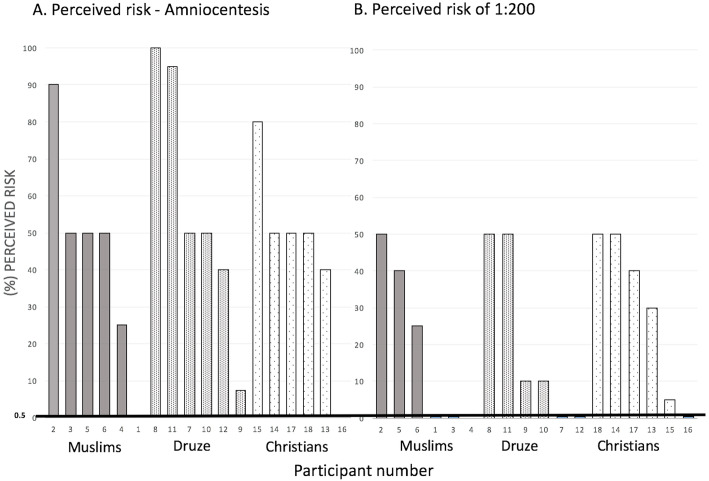
Risk perception amongst individual interview participants. **a** Participants were asked to indicate their perceived risk of amniocentesis. This was entirely based on prior knowledge of the test. They were not provided with any additional information to skew their response. **b** Interpretation of a hypothetical risk of 1 in 200 births (1:200) for Down syndrome. Patients indicated their understanding of this 1:200 risk with a mark on a horizontal line representing 0–100% chance of Down syndrome. Only participants who successfully completed these tasks are included (some did not perform one or both of the tasks at all). Objective risk is indicated with black line at the value of 0.5%

We next wanted to focus on patient comprehension and perceptions of genetic risk as communicated during counseling sessions (see Additional file 1, Communication of genetic risk). Amniocentesis was perceived as being very risky (more than a 50% perceived risk) by most of the participants (Fig. 1). In reality, the most common complication of amniocentesis is contamination that can lead to miscarriage, with an estimated risk of less than 0.5% [44]. Other potential injuries to the baby or mother are extremely rare [44]. Thus, the 50% risk stated by most participants is a vast overestimation. One of the participants explained the threat was due to the Arabic colloquial name for the procedure: “*mayte al ra’as*” meaning “water off the head of the fetus.”

Similar to the risk of amniocentesis, women tended to perceive a risk of 1:200 for Down syndrome as being much higher than the objective risk of 0.5% (Fig. 1. Mean for all groups: 25.75%, SD 21.36). The gap between the actual risk of this hypothetical scenario and the perceived risk as expressed by the participants can be interpreted in several ways—either counselor- or counselee-dependent or both—but all imply a startling failure of communication between the two sides during counseling.

### Similarities and differences in HCPs versus genetic counselee perceptions

We next compared responses from individual counselee interviews and those from the HCP focus groups including counselors and nurses to identify general thematic similarities and differences (Table 3). HCPs’ opinions regarding the previously discussed themes were assessed, and additional differences were identified, particularly between counselors (as opposed to nurses) and counselees.

**Table 3:** Comparison of responses from individual interviews and focus groups

		Genetic counselors	Nurses	Counselees
**Lack of knowledge regarding genetic counseling**	Lack of pre-counseling knowledge	Aware of counselee lack of knowledge	Aware of counselee lack of knowledge	Pervasive lack of knowledge
	Desire for action - need for some type of community education	Did not mention	Suggested “marketing” campaign in the community	Desire for community outreach
**Negative personal and cultural preconceptions about genetic testing and counseling**	Lack of trust	Mention lack of trust from counselees	Mention lack of trust and negative narrative	Some negative narrative
	Referral interpreted with stigma	Aware of stigma	Aware of stigma	Fear of stigmatization
	Misconception - expectation for negative event	Did not mention	Aware of counselee’s negative pre-conceptions	Negative pre-conceptions
**Lack of effective communication regarding the counseling service**	Risk perception	Objective based- Provide technical definitions statistics	Counselees interpret as dichotomous	Counselees interpret as dichotomous
**Culturally-influenced differences in decision making regarding pregnancies between Arab subsets**	Family involvement	Feel all families can interfere	Aware of Muslims family interference	- Muslims: family interference - Christians: family support - Druze: Did not mention
	Termination of pregnancy	No distinction between Arab subgroups	Aware of Muslim and Druze objection due cultural reasons	Most Muslim and Druze object for reasons of conscience and culture
**Other differences between counselor and counselee perceptions**	Major expectation for genetic counseling outcome	facilitator of informed consent	Teach genetic information	Desire for empathy and reassurance from counselors
	Language	Major barrier to effective counseling	Mild mention of language barriers	No mention of language as barrier

Regarding knowledge and preconceptions about genetic testing and counseling, nurses and counselors recognized the general lack of understanding about the genetic counseling profession by their counselees and the negative preconceptions they brought with them (Table 3). Counselors noted that counselees had no idea what to expect when they arrived for counseling sessions. For example:

*“... They [Arab counselees] do not know where they come to, or they come to the clinic, thinking [that] I'm going to do something, ... or I do not know what, like do a physical examination …”*

Counselors expressed some frustration regarding the probably insufficient explanation provided to the patients prior to genetic screening tests and the likely insufficient initiative on the part of counselees to ask questions.

*“The first problem is that 90% of women perform the test, and they don't know what they did… as far as they are concerned, they did a blood test, and it's a very, very big obstacle ...”*

This corresponded with the frequent confusion expressed by counselees between genetic testing and ultrasound (commonly assumed to be “better” than genetic testing; Table 2, 2c), not understanding that they are two distinct types of tests with potentially different findings. The stigma against genetic counseling was recognized by the counselors, as many in the focus group emphasized the apprehension of counselees about sharing information regarding family medical history.

*“There are illnesses in the family, and they are not able to bring the information, they cannot ask why there are three sick cousins with the same illness .... as if they should not ask, [it is] unpleasant, not respectful and you are stuck... and I feel stuck because they are cousins, his uncle has three sick children, I have no idea what it is. ... I feel like I have no way of achieving it ... and no way to help the family …”*

Despite acknowledging these issues, counselors made no mention of strategies for increasing public awareness, yet counselees and nurses expressed the need to reduce stigma and negative preconceptions (Table 3).

*“First of all, marketing that it (genetic counseling) is not scary, is not so threatening, it is when you come with your husband … it is something like a nurse working at the station they are used to ...” (Druze nurse)*

As for the explanation of risk, counselors described a variety of technical approaches to explaining the statistical data available about genetic risk to motivate counselees to act (Table 3).

All participants focused on describing technical methods for clarifying the relevant information. However, all the counselors used different analogies, such as:

- *“Of 100 balls in the basket, only one is afflicted … I stress that 199 are safe.”*
- *“I use a rectangle drawing. I illustrate to the woman that I am only referring to the part of the marked rectangle.”*

The nurses group explained the overestimation of risks as a dichotomous interpretation amongst the counselees, meaning they did not understand or care about the range of potential risks and merely wanted to know if their pregnancy was normal or abnormal (Table 3). In their words, *“an abnormal biochemical test for them means a fetal malformation.”* This corresponds with our prior observation that the counselees tended to overestimate risk (Fig. 1) because, from their perspective, any risk at all is still a risk for potential abnormality.

Several other differences between counselors and counselees arose in our comparison*.* First, while many counselees and nurses emphasized the significance of the counselor–counselee relationship and desire for empathy, genetic counselors were more focused on relaying objective information in the most unbiased way possible (Table 3) and depicting the need to address psycho-social issues related to genetic counseling as being part of the social worker’s role. In their words:

*“Now we have a social worker, so that if there are [psycho]-social issues, she is involved as well ... [but] she works [only] 4 days per week, 08:00-13:00. So that's it, we find ourselves doing it (providing emotional support), we don't have the time and means [professional training] to do so.”*

Second, the counselor group made extensive references to the difficulty in communication, with language being one specific barrier, although the counselees did not recognize this as an issue (Table 3). Demographically, counselors in our focus group were mainly Jewish and often did not speak Arabic. Thus, they were probably more sensitive to language issues than the Arabic-speaking nurses. We shall refer further to the importance of the ethno-cultural affiliation of the HCPs in providing more ethical and effective service to counselees of minority cultural groups in the Discussion section that follows.

Third, counselors tended to overestimate the influence that spouses had on pregnancy decisions (Table 3), particularly amongst their Druze and Muslim patients, while our data (Table 2) indicated that, in the majority of cases, the women felt that they maintained autonomy of decision-making in such matters.

*“... I do not know Arabic, the husband sits in the middle, what he is translating her, or what he is translating from her, if there is a connection between things I do not know, I hope so. ... We need a translator. ... Well, in extreme cases, we have help from the team ...”*

Finally, counselors did not distinguish between Arab subsets with regard to their expectations and culturally competent counseling approaches, particularly pertaining to family involvement and termination-of-pregnancy decisions, despite the major differences identified herein. However, they did feel that counselees with a certain education level were more likely to be involved.

*“The population in Haifa is different from the population in villages. The population in Haifa is more educated … (whereas with the population from teh) villages there are language and cultural difficulties ...”*

Nurses were more attuned to differences among their Muslim, Druze, and Christian patients. Their comments often more closely depicted the general themes expressed during the individual counselee interviews (Table 3). These differences resulted in a general lack of trust and ineffective discourse, as one frustrated counselor described:

*“There is a lack of trust ... because all of the stories... (they) are already 'anti' … unwilling to hear counseling related to invasive tests and so on. … Consulting has become bland; you say what you have to say. ... They no longer have the ability to listen. … It seems to me they come only to sign an attendance card…”*

## Discussion

Our study addresses and highlights three main important themes related to a more nuanced understanding of cultural competence in the context of genetic testing and counseling for minority ethnic and cultural groups. In what follows, we shall elaborate on each of these themes while referring to relevant existing literature and finally connecting them to the issue of autonomy in genetic testing and counseling for minority ethno-cultural groups.

### Potential gaps between majority group counselors and minority group counselees

Our study has revealed fundamental miscommunications and gaps in the expectations between counselors and counselees, which engenders distrust and frustration with the counseling process on the part of both parties. These miscommunications and gaps between the two groups pertained to the following three main facets: (a) communication approaches, whereby counselors were more focused on the presentation of objective aspects of genetic risks and verbally expressing them in a comprehensible manner while counselees were more expectant of definitive messages accompanied by emotional reassurance and connection with counselors; (b) comprehension and perception of genetic risk counselees, whereby they overestimated the meaning of the cited genetic risks; and (c) estimation of spousal influence, in which counselors expressed an overestimation of the spousal role and influence, especially among Muslim and Druze counselees, compared to counselees’ reference to this issue.

Other studies have already shown possible differences in attitudes toward genetic testing between minority groups and the majority group within the same country. For instance, some studies in the USA have noted that Caucasian people and those with Hispanic and Asian origins have more positive views toward genetic tests compared to African-Americans [45–49]. A more recent study referring to ethno-cultural minorities with a preference for consanguineous marriages in the Netherlands described the attitudes and awareness of Dutch Moroccans and Turks regarding consanguinity and its associated reproductive risk [50]. Other studies focusing on the attitudes toward genetic testing among medical students deliberately focused on minority ethno-cultural groups precisely due to the assumption that has been indeed confirmed that such groups may have a different perspective on genetic testing than the majority group. Such studies focused on either a minority group within developed countries (e.g., African-American medical students in the US) or non-Western developing regions (e.g., Sub-Saharan Africa, East Asia), constituting a “minority” perception compared to the Western hegemonic view [51–54].

However, our study is the first to focus on actual gaps between patients (or counselees, in this case) from a minority group and HCPs (in particular, genetic counselors) from the majority cultural group regarding the process of genetic testing and counseling. One possible reason for this persisting miscommunication is that the genetic counselors were entirely comprised of the Jewish majority group. Consequently, it could be argued that the interaction between counselors and counselees was reflective of the overall majority–minority interaction in the country as a whole, regardless of the fact that the Arab population in the Western Galilee geographic regions are the majority. Indeed, the nurses in our study originated from minority subgroups and, unlike the counselors, spoke Arabic and recognized both the counselor and counselee perspectives on many of the points presented herein—perhaps due to being part of both ethnic worlds. They proved their extreme usefulness as liaisons between the two parties.

### Deeper and more complex understanding of factors underlying reluctance to receive counseling

Our study further stresses the possible negative sentiments among minority ethno-cultural and religious groups in a manner that was not exposed before. For instance, two previous studies in Israel demonstrated the underutilization of genetic counseling services among Arab women compared to the Jewish majority, concluding that the reasons were lower income level, negative attitudes toward genetic counseling, perception of amniocentesis as risky, spousal opposition to the process, and poor accessibility [27, 28]. Previous international studies have also observed that genetic counseling is associated with many negative psychological feelings and social stigmatization [55–57]. In these studies, negative sentiments, such as shame, were discussed in association with a diagnosis of genetic diseases in general or with regard to disability.

However, the current study uncovered aspects of negativity toward genetic counseling among Arab counselees that were not depicted as vividly before. Thus, we observed that even the act of referral to genetic counseling services carries a great risk for ignominy amongst our study populations. In addition, we found a drastic overestimation of risk among the Arab populations regarding amniocentesis as well as the interpretation of relative risk as much higher than objectively specified. As the hypothetical scenarios used in our study closely mimic those that occur in many counseling sessions, a similar lack of communication of essential knowledge regarding genetic risk and the tests involved in genetic counseling is likely common in the community. In fact, previous studies have already described a lack of knowledge and awareness of genetic testing and counseling [18, 58, 59]. It has also been shown that individuals with more informed prior knowledge of genetic counseling sessions have better outcomes [60]. Moreover, while overestimation of risk is mentioned in other studies of counseling sessions [18, 38, 61], the current study uncovers a possible specific cultural influence underlying such an overestimation. Hence, the extensive overestimation in the Arab culture could be due to the Arabic phrase “water off the head.” One can imagine how such a description could imply a greater threat to a fetus than actually exists when the fetus itself is not tested.

At the same time, our study highlights possible complexities in the attitude of counselees from the minority Arab group toward genetic testing and counseling. As noted herein, many of our interviewees exhibited negative attitudes, suspicion, and a lack of understanding regarding genetic counseling and its meaning. Yet they also expressed a desire to be more informed and educated regarding this subject. This tension between the negative attitudes toward genetic counseling and the desire to be more informed about it echoes similar tensions already observed in other studies involving cultural underpinnings in healthcare provision. Therefore, this tension supports the culturally driven lack of communication between genetic counselors and their counselees, as will be further elaborated in the next subsection.

### Differences between subcultural groups within minority ethno-cultural groups

We also uncovered important differences between Arab subsets regarding the desire for family involvement and termination of pregnancy—differences largely overlooked by the counselors themselves. These areas of contrast between the varied cultural subsets within the Arab minority group could greatly influence who should be included in genetic counseling discussions and what goals or expectations should be addressed. These issues are currently not being effectively addressed by the counseling services, especially the genetic counselors who appeared to perceive the Arab counselees as one monolithic group. Indeed, other cultural or religious subgroups within populations vary on similar topics [62]. Thus, culturally competent counseling practice should address such issues early on in the process.

Furthermore, the importance of acknowledging the viewpoints of cultural subgroups within minority groups is emphasized when linking our results to the “process of cultural competence” model, a much-cited model about cultural competence by Campinha-Bacote. A key feature in this model is the idea of cultural awareness, which relates to the ability of the individual HCP, including genetic counselors, to acknowledge their *own* cultural underpinnings and not merely the influence of culture on their patients’ perceptions [63]. Cultural awareness is crucial to the overall cultural competence of health care providers, because “without being aware of the influence of one’s own cultural or professional values, there is risk that the health care provider may engage in cultural imposition” ([63] , p. 182). One such imposition may be the viewing of minority cultural groups in a monolithic manner, especially from the perspective of members of the majority group, as in the case of the genetic counselors in our study. In fact, overall, training for cultural competence, including its entailed cultural awareness, has been found to be important at both the public health level and the individual-based patient–healthcare professional contact and communication level [64–67].

### Importance of culturally competent HCPs for empowering personal autonomy

Thus far the discussion has illuminated, from different perspectives, the possible cultural underpinning of the gaps in the concept of genetic testing and counseling between genetic counselors from a majority group and counselees from a minority group. Based on this discussion, the importance and lack of cultural competence among Israeli HCPs was stressed. However, the question still remains how enhancing the cultural competence of HCPs, whether in Israel or other countries, may be related to overcoming the gap between the goal of genetic counseling to empower the autonomy of counselees and the perception of minority groups, like the Arab minority in Israel, regarding genetic testing and counseling as curtailing their autonomy. In this final portion of the Discussion section, we offer one possible answer based on the theory of cultural value orientation developed by Schwartz.

According to Schwartz’s theory, three main challenges that every cross-cultural society faces can be handled by bipolar value-based cultural viewpoints, thereby creating value-based conflicts in handling these challenges [68]. One such conflict pertains to the issue of seeing how people manage their relationships with the natural and social world, in which one end of the cultural spectrum stresses the value of harmony whereas the other end proclaims the value of mastery. The latter culturally based value is defined as the “active self-assertion in order to master, direct, and change the natural and social environment to attain group or personal goals” ([68] , p. 141). Hence, emphasizing mastery could be aligned with cultures that stress personal autonomy in the sense that the individual is her own master. But such stress on the value of mastery may also result in a cultural or societal viewpoint that does not give space for other perspectives because the focus is on mastering or directing the natural and social environment to attain the specific social and cultural goals to which the group subscribes.

This latter facet of asserting the value of mastery is possibly being echoed in the Jewish genetic counselors’ insufficient understanding of the cultural complexities underpinning their genetic counselees from the Arab minority group. Instead of being sensitive to these cultural complexities and nuances as described in our study, the genetic counselors from the Jewish majority group seem to be focused on the delivery of the “objective” Western scientific or medical results. This sort of communication, as we have seen, may result in miscommunication and distrust from the counselees from the minority group toward the counselors from the majority group, including the perception that genetic testing and counseling are untrustworthy or alternatively compel counselees to perform abortions against their will.

In other words, the same value (i.e., mastery) that is supposed to entail respect for personal autonomy in genetic counseling seems to also underlie the insufficient sensitivity to the cultural complexities underpinning the viewpoint of genetic counselees from the Arab cultural group (or one of its subsets). On the other hand, harmony, the contrasting cultural value, seems to be related to a cultural viewpoint that cherishes cultural awareness as part of efforts to foster *cultural competence.* Indeed, the value of harmony is described as “fitting into the world as it is, trying to *understand and appreciate rather than to change, direct*, or to exploit” [68, p. 141 (emphasis added)]. Therefore, we argue that, by fostering a more culturally sensitive perspective, HCPs from the majority (Jewish) group, such as the case of the genetic counselors in our study, may find a better balance between mastery and harmony values. Such a balance, in turn, may assist these HCPs in genuinely attaining the goal of genetic testing and counseling through the empowerment of personal autonomy of counselees from minority group(s).

### Study limitations

As with all qualitative studies, our results are based on interviews with a relatively small number of participants. Thus, generalizability is always a concern. We did not include a non-Arabic interview group, such as the majority Jewish population in Israel, as a control to highlight minority-specific issues. However, other studies referenced in the Discussion section that do compare results with the Jewish majority support our conclusions. Moreover, the counselors and nurses were able to provide some insights into Arab-specific issues due to their experience caring for both populations, and similar results have been found for other minority populations around the world (see the Discussion section).

With respect to the method of performing the analysis, we acknowledge that it is often suggested to perform an inter-rater reliability test, with two researchers independently analyzing the data. However, in our research group, only the first author is a professional genetic counselor; thus, we preferred for this author to perform the main analysis while engaging in an ongoing discussion with the second author in order to ensure the trustworthiness of the study. Indeed, in cases where two independent analyses are less feasible, such an approach has been used in other publications and deemed acceptable [69–71].

Finally, a few confounding factors were not addressed, such as differences in levels of education, employment status, occupation, social isolation, and level of religiosity between the Arab subsets, all of which may have influenced the extent to which participants were exposed to health-related information. However, as the current study is qualitative rather than quantitative, it is beyond its scope to explore possible confounding factors. Indeed, future statistically based quantitative studies in this domain may further explore the potential influence of these confounding factors. In addition, the major differences that we observed between groups, particularly regarding family involvement in decision-making and termination-of-pregnancy decisions, were largely based on cultural and religious grounds and were thus unlikely to be dependent on occupation. The degree of religiosity may impact pregnancy decision-making; therefore, comparisons of religious versus non-religious groups among minority and majority populations may reveal impactful results.

## Conclusions

Acknowledging and better understanding identified barriers to genetic counseling in the Arab subpopulations are likely to help decrease gaps in the utilization of genetic counseling, improve the multicultural competence of counselors and their services in general, and enable more effectively tailored interventions to serve this large ethno-cultural and religious minority group better within Israel and many other countries. Such an understanding may also genuinely empower the personal autonomy of counselees from the Arab minority group and its applicable subsets, thereby potentially better achieving a key goal in the Western approach of non-directive counseling, whether in Israel or in other countries with this minority group.

## Supplementary information

**Additional file 1.** Communication of genetic risk.

## Data Availability

The datasets generated or analyzed during this study are not publically available since they use foreign language. However, the datasets are available from the corresponding author on reasonable request.

## Abbreviations

GC: Genetic Counseling
NSGC: National Society of Genetic Counselors
