# Supplementary material for: Challenges to effective and autonomous genetic testing and counseling for ethno-cultural minorities: a qualitative study
Source: BMC Med Ethics. 2020 Oct 15;21:98. doi: 10.1186/s12910-020-00537-8 (PMC7565773; doi:10.1186/s12910-020-00537-8)
Supplement: Supplementary file 1 — Additional file 1. Communication of genetic risk. [file 12910_2020_537_MOESM1_ESM.docx]

**Appendix 1 – Communication of genetic risk.**

- A risk of 1:200 for Down syndrome was given to your friend.

(Mark your risk perception of 1:200).

100% affected _____________________________________________ no risk

- The GC offered your friend prenatal diagnosis - (amniocentesis )

What is the risk for amniocentesis? (Mark your risk perception).

100% abortion_____________________________________________ no risk
